# The New Strategy for Studying Drug-Delivery Systems with Prolonged Release: Seven-Day In Vitro Antibacterial Action

**DOI:** 10.3390/molecules27228026

**Published:** 2022-11-18

**Authors:** Anna A. Skuredina, Tatiana Yu. Kopnova, Anastasia S. Tychinina, Sergey A. Golyshev, Irina M. Le-Deygen, Natalya G. Belogurova, Elena V. Kudryashova

**Affiliations:** 1 Department of Chemistry Department, Lomonosov Moscow State University, 119991 Moscow, Russia; 2 Belozersky Institute of Physico-Chemical Biology, Lomonosov Moscow State University, 119991 Moscow, Russia

**Keywords:** antibacterial activity, in vitro, fluoroquinolones, cyclodextrin, TEM, adsorption, particle adsorption visualization

## Abstract

The new method of antibacterial-drug-activity investigation in vitro is proposed as a powerful strategy for understanding how carriers affect drug action during long periods (7 days). In this paper, we observed fluoroquinolone moxifloxacin (MF) antibacterial-efficiency in non-covalent complexes, with the sulfobutyl ether derivative of β-cyclodextrin (SCD) and its polymer (SCDpol). We conducted in vitro studies on two *Escherichia coli* strains that differed in surface morphology. It was found that MF loses its antibacterial action after 3–4 days in liquid media, whereas the inclusion of the drug in SCD led to the increase of MF antibacterial activity by up to 1.4 times within 1–5 days of the experiment. In the case of MF-SCDpol, we observed a 12-fold increase in the MF action, and a tendency to prolonged antibacterial activity. We visualized this phenomenon (the state of bacteria, cell membrane, and surface morphology) during MF and MF-carrier exposure by TEM. SCD and SCDpol did not change the drug’s mechanism of action. Particle adsorption on cells was the crucial factor for determining the observed effects. The proteinaceous fimbriae on the bacteria surface gave a 2-fold increase of the drug carrier adsorption, hence the strains with fimbriae are more preferable for the proposed treatment. Furthermore, the approach to visualize the CD polymer adsorption on bacteria via TEM is suggested. We hope that the proposed comprehensive method will be useful for the studies of drug-delivery systems to uncover long-term antibacterial action.

## 1. Introduction

The development of novel antibacterial-drug formulations and drug-delivery systems (DDS) is a vast research area [1,2]. Such studies mainly cover the physical and chemical properties of the formulation, for instance, its structure, the type of drug-carrier interactions, drug release in buffer solution, etc. [3,4,5]. However only a few papers address the drug’s antibacterial effect upon incorporation into the DDS [6,7].

Most of such in vitro investigations focus only on how the DDS affects the drug’s minimum inhibition concentration (MIC) or its antibacterial activity. It is important to note that methods used in these studies can be applied only for a limited period of time: usually 24–48 h [8,9,10,11,12]. However, some formulations show sustained drug release for up to 10 days [13,14,15]. That means the whole potential of drug formulation cannot be investigated in 24–48 h.

In the literature, the long-term study of antibacterial activity is considered, but research includes in vivo studies or is conducted using eukaryotic cells [16,17]. For instance, the authors in [18] studied tobacco bacterial wilt on plant seedlings using *Ralstonia solanacearum* for 7, 14 and 21 days. Prokaryotic cells draw the most attention only when they grow slowly: for example, *Xylella fastidiosa*’s became visible on agar plates only after 5–7 days of incubation [19]. However, the majority of bacterial strains grow faster, which limits the time of classical methods in microbiology.

Hence, the new approaches are essential for the comprehensive study in vitro of a drug’s antibacterial action over longer periods. In this paper, we developed a novel strategy for an antibacterial 7-day in vitro study, using polymeric cyclodextrin particles as the carriers that demonstrate sustained and prolonged drug release.

Among the number of DDS cyclodextrins (CDs), torus-shaped oligomers of glucose are widely used, due to their low-cost, biodegradability, and the ability to form non-covalent «guest-host» inclusion complexes with hydrophobic organic molecules [20]. The complex formation improves the drug’s physical and chemical properties such as solubility [21], bioavailability, and stability [22]. CD derivatives, including oligomers and polymers obtained by crosslinking CDs, as well as their complexes with drugs, are comprehensively described in the literature. Nevertheless, it is still a marvelous challenge to uncover the effect of CDs on a drug’s antibacterial action.

We chose fluoroquinolone moxifloxacin (MF) as a model drug that can form complexes with CD derivatives, as we have shown previously [4,23]. MF is used against both gram-negative and gram-positive bacteria. Moreover, COVID-19 pandemic fluoroquinolones can be reasonably used for the treatment of bacterial secondary infection—COVID-19-associated pneumonia [24,25]. In addition, many authors report limited antiviral activity of fluoroquinolones against SARS-CoV-2 [26]. In this study, the 7-day antibacterial activity of MF complexes with CD and its polymer is investigated by the proposed experimental approach. Furthermore, we discuss the correlation between the complexes’ parameters (size, dissociation constant, release profile, etc.) and their antibacterial activity in vitro. This newly outlined strategy is promising for the deep in vitro study of novel-drug formulation, especially drug delivery systems with a prolonged release.

## 2. Results

**Figure 1:**
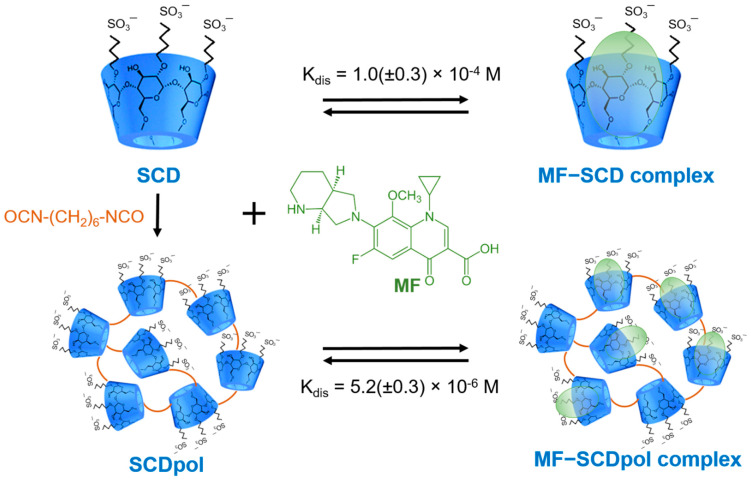
The scheme of obtained MF complexes with SCD and SCDpol.

As DDS we chose sulfobytul ether β-CD (SCD) and SCD-based polymer (SCDpol), branched polyurethane, obtained by the crosslinking of SCD via 1,6-hexamethylene diisocyanate (Figure 1). NTA analysis showed that SCDpol forms nanoparticles, with an average hydrodynamic diameter of 180–200 nm and Mr~140 kDa (Table 1). Due to the presence of the sulfo groups, both SCD and SCDpol possess negative ζ-potential (–7.3 ± 0.8 mV and –18.7 ± 1.1 mV, respectively). The K_dis_ values of MF-carrier complexes are 1.0 (±0.3) × 10^−4^ M and 5.2 (±0.3) × 10^−6^ M, respectively. The physical-chemical properties of the studied compounds are summarized in Table 1. The difference in size and stability between MF-SCD and MF- SCDpol might affect the resulting antibacterial activity of the DDSs.

We have recently studied CD polymers and their «guest-host» complexes with the fluoroquinolone drug MF, using FTIR, NMR and circular dichroism [23]. The main binding site of MF is the CD cavities. The 20-fold increase in the complex formation effectiveness in the case of the SCDpol carrier might be associated with additional interactions between MF and the hydrophobic chains of the linker in the polymer matrix. Furthermore, SCD polymers demonstrate the tendency to prolong the MF antibacterial activity. Here, we study this phenomenon in depth, by varying the drug concentration and bacteria strain, and investigate how SCD affects the MF mechanism of action and adsorption of bacteria.

### 2.1. Antibacterial Action of Moxifloxacin against Two Strains of Escherichia coli

**Figure 2:**
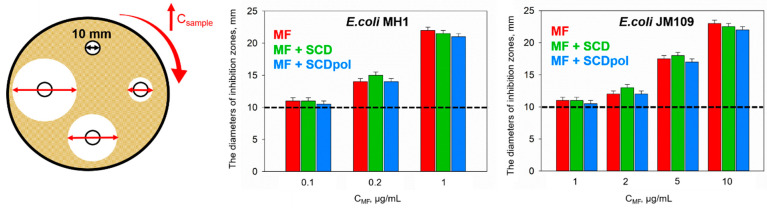
The resulting inhibition zones (DIZ) on Petri dishes after the introduction of MF and its complexes with SCD and SCDpol, using the agar diffusion method, C_MF_ = 0.05–10 μg/mL, pH 7.4 (0.01 M PBS), 37 °C, 24 h of incubation.

*E. coli* strains are commonly used for antibacterial studies [7,27,28]. First, we determined the minimum inhibition concentration (MIC) of the samples on two *E. coli* strains, *E. coli* MH1 and *E. coli* JM109, using the agar-well diffusion method (Figure 2 and Figure S1), which is a fast and robust approach [6,12,29]. The *E. coli* MH1 and *E. coli* JM109 strains differ significantly in cells surface-structure, which is a crucial bacteria characteristic as it might affect the drug adsorption and, as a consequence, its antibacterial action.

MF’s antibacterial action demonstrated a pronounced dose-dependence (the diameters of the inhibition zones (DIZ) depend on the drug concentration), for both strains. The MIC values for *E. coli* MH1 and *E. coli* JM109 were 0.1 μg/mL and 1 μg/mL, respectively (Figure 2). These values are in good agreement with fluoroquinolones’ antibacterial activity against other gram-negative bacteria [30]. Fluoroquinolones enter the cell by passive diffusion, so the ten-fold lower sensitivity of *E. coli* JM109 compared with *E. coli* MH1 might be explained by the differences in the surface morphology of the bacteria surface and/or gyrA96 mutation (Table S1).

As expected, neither SCD nor SCDpol demonstrated any antibacterial effects, as CDs have been repeatedly proved to be non-toxic and biodegradable [8,31]. MF complexes with CDs have comparable antibacterial activity to that of the free MF.

**Figure 3:**
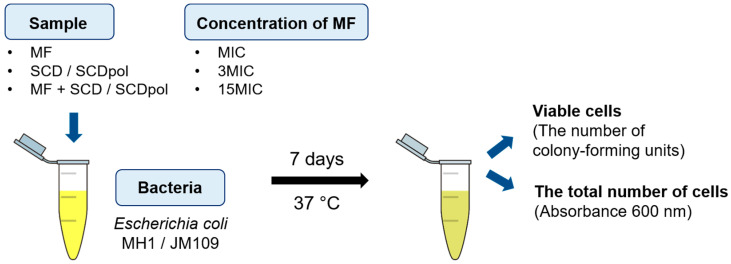
The scheme of seven-day in vitro experiment in liquid media, 37 °C.

To analyze the prolonged antibacterial action, we designed the experiments in liquid media (Figure 3). The bacteria growth-conditions were specially selected so that the cells remained viable for a long time. MF, MF-SCD, and MF-SCDpol were added to the cell culture-medium to obtain the MIC and the triple MIC and fifteen-fold excess of MIC (3MIC and 15MIC, respectively). The cells were incubated with the samples for seven days. At a given time, the aliquots of the cell suspension were seeded onto the Petri dishes for the quantitative assay of viable cells—colony-forming units (CFU). The absorbance at 600 nm [32,33] was used to monitor the total number of cells and the cell lysis.

The control cultures of both bacterial strains were grown for 3–4 days; the absorbance at 600 nm remained constant (no lysis was detected), and the number of CFUs decreased (Figure S2).

**Figure 4:**
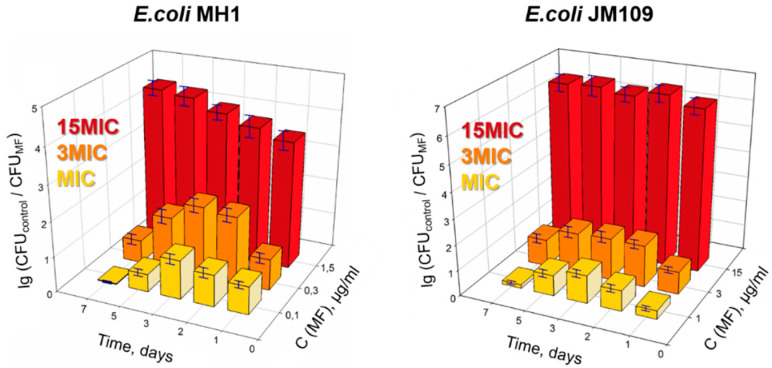
The MF’s antibacterial effect at three different concentrations (MIC, 3MIC, and 15MIC) in a seven-day experiment in liquid media, on *E. coli* MH1 and *E. coli* JM109, pH 7.4, 37 °C.

As in solid-media experiments, MF had a pronounced dose-dependent antibacterial effect against *E. coli* (Figure 4). At MIC and 3MIC, the drug demonstrated antibacterial activity during the first 3 days; however, afterward the MF stopped working. At 15MIC, MF demonstrated a noticeable effect throughout the experiment. The absorbance at 600 nm of all the samples showed the same trend as the CFU.

**Figure 5:**
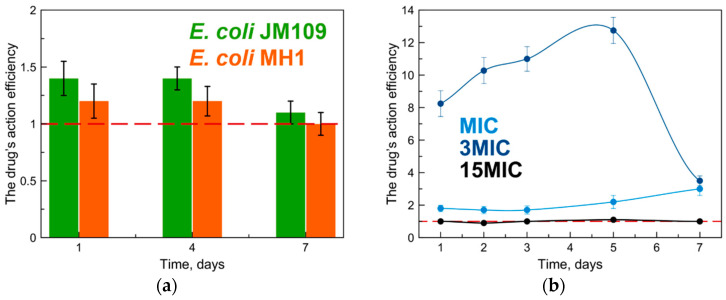
(**a**) The values of the drug’s action efficiency (DAE) for MF–SCD, C_MF_ = 0.1 μg/mL for *E. coli* MH1 and 1 μg/mL for *E. coli* JM109, pH 7.4, 37 °C. (**b**) The values of the drug’s action efficiency (DAE) for MF–SCDpol on *E. coli* JM109, C_MF_ = 1 μg/mL, 3 μg/mL or 15 μg/mL, pH 7.4, 37 °C. The red dashed-line demonstrates MF’s action under the same conditions.

As in the experiments in the solid media, SCD and SCDpol themselves did not exhibit an antibacterial effect on both strains (C_CD torus_ < 10 µg/mL): the CFU counts and absorption at 600 nm were comparable to the appropriate values for the control samples of bacteria (Figure S2). The formation of MF complex with SCD affected the antibacterial properties of MF only in the first four days: the values of the drug’s action efficiency (DAE) were in the range of 1.2–1.4 (Figure 5a). On the seventh day, the complex demonstrated antibacterial activity comparable to the activity of the free drug, i.e., no prolonged action was observed for MF–SCD. The low stability of the MF-SCD complex (K_dis_~10^−^^4^ M) might maintain MF’s protection only for the first 3–4 days.

We observed completely different results for MF–SCDpol (Figure 5b). This sample was characterized by K_dis_~10^−^^6^ M. The polymer carrier dramatically enhanced the MF’s antibacterial effect from the first day of the experiment (at concentrations close to MIC). The investigation at high drug-concentration (15MIC) demonstrated an effect comparable to free MF (DAE~0.9 ± 0.4).

The highest drug’s action efficiency (DAE) value was 12 ± 0.9, for *E. coli* JM109. For the *E. coli* MH1 strain, an increase in the antibacterial activity of MF was also observed, but the DAE had lower values (up to 3). The differences in bacteria surface-morphology might be assumed to be the primary reason for this.

Interesting, one can observe the sharp DAE decline on the seventh day for 3MIC. Since we did not observe this effect for MF at MIC, the biodegradation of the drug observed previously for both MIC and 3MIC is unlikely. We suggest that this effect might be associated with the retention of the remaining MF molecules in the SCDpol matrix that was used at the higher concentration in the case of 3MIC. Nevertheless, this statement must be investigated.

Moreover, the presence of the drug and the absence of a new growth-medium caused the considerable decrease in CFU on the seventh day (Figure S2, Figure 4 and Figure 5). Under such conditions, remaining viable cells might show changes in metabolism and act differently, so we believe that the proposed experiment is limited to 7 days.

To sum up, the CD polymer particles significantly improved the antibacterial properties of the drug. This effect may be associated with the enhanced adsorption of CD particles on the cell surface resulting in a high local concentration of the drug. Moreover, CD adhesion can cause membrane damage [11]. In addition, CD-based carriers can interfere with MF biodegradation, meaning that the drug’s incorporation into the polymer matrix may protect the active molecule from enzymatic and chemical influences.

### 2.2. TEM Studies of Bacteria State during a 7-Day In Vitro Experiment

**Figure 6:**
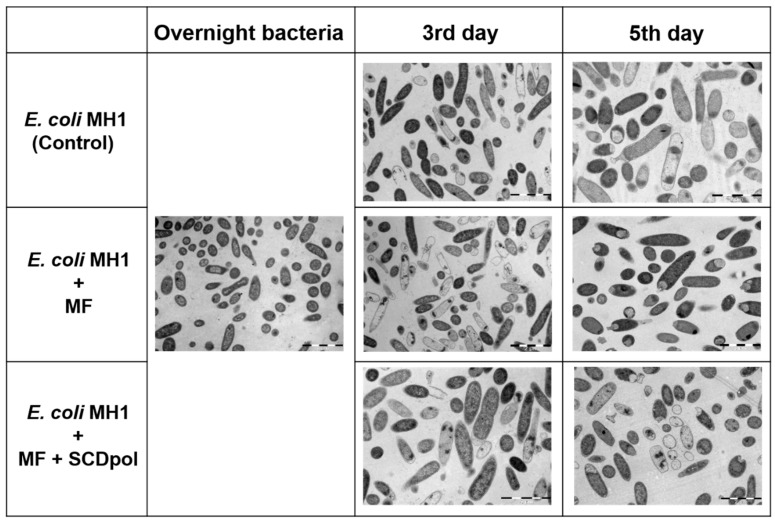
The TEM micrographs of the Escherichia coli MH1 strain. Overnight culture; cells incubated independently or with the drug formulation at MIC in liquid media for 3 or 5 days, 37 °C. Scale bar—2 μm.

The development of the method for studying prolonged antibacterial action requires an in-depth study of the bacteria state during the long-period experiment. We conducted the ultrastructural studies using transmission electron microscopy (TEM), which is a powerful method for the determination of the bacteria state (Figure S3). The overnight culture’s ultrastructure demonstrated a homogeneous bacteria-state (Figure 6). We observed no difference between the morphology of *E. coli* MH1 and the *E. coli* JM109 strains (Figure S4). The incubation of bacteria for 3 and 5 days increased the morphological heterogeneity. Although many dead cells were detected, the majority of the bacterial cells retained typical structural features (Figure 6).

On the micrographs of bacteria exposed to MF, MF–SCD, or MF–SCDpol for three days, the number of dead cells is higher, compared with the control sample. Nevertheless, most of the cells remained alive. The prolongation of the exposure for two more days led to the increase in homogeneity for the sample containing MF, whereas the MF-SCDpol action was comparable to the third day. This proves our recent data on the loss of MF’s antibacterial activity when working at near-MIC values. MF, MF–SCD, and MF–SCDpol did not affect the morphology of the cell wall, membrane or cytoplasm.

### 2.3. The Surface Morphology of E. coli MH1 and E. coli JM109

**Figure 7:**
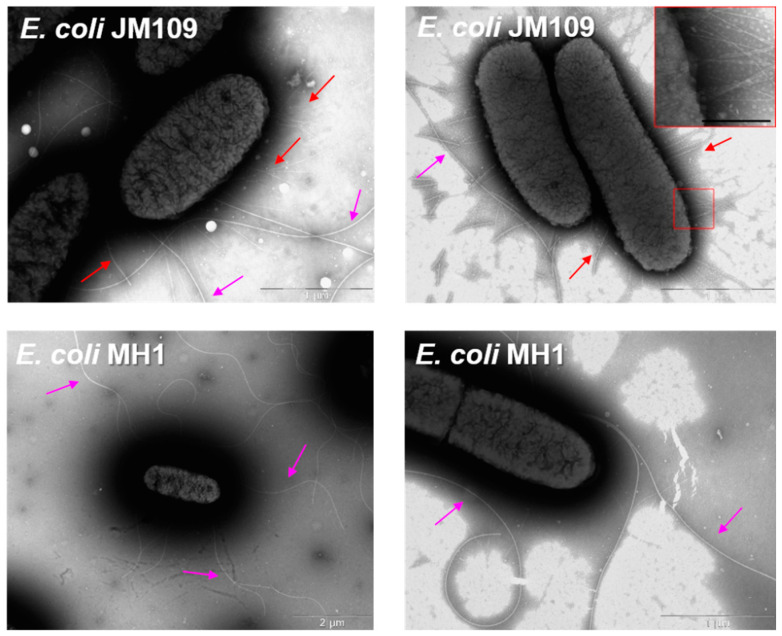
The morphology of the *Esherihia coli* MH1 and JM109 bacteria cells. Pink arrows indicate flagella and red arrows are for fimbriae. Scale bars—1 μm, 1 μm, 2 μm and 1 μm, respectively.

We studied the absorption of the particles on the bacteria surface, as could not be observed via ultrastructural studies (Figure 6), due to the sample preparation-procedure; we used negative staining to uncover the morphology of the bacteria. The TEM micrographs of *E. coli* are shown in Figure 7. Both *E. coli* MH1 and *E. coli* JM109 have a few long flagella (~2.5–4 μm in length) on each cell. In addition, all the *E. coli* JM109 surface is covered with numerous protein outgrowths of fimbriae (~0.3–1 μm in length).

Furthermore, the difference in the surface morphology might demonstrate a higher adsorption of the drug formulation and, as a consequence, the higher antibacterial action on *E. coli* JM109.

### 2.4. ζ-Potential of E. coli Cells in the Presence of MF-SCD Complexes

The possible mechanism of MF-SCDpol high antibacterial action might be the particle adhesion on the cell surface. The polymer adhesion on the cell membrane is considered to act primarily via electrostatic interactions.

*E. coli* MH1 and *E. coli* JM109 possess a pronounced negative ζ-potential of –36.8 ± 4.5 mV and –35.5 ± 5.6 mV (in MilliQ H_2_O), respectively, which is in good agreement with the literature data for *E. coli* strains [34,35]. The values of the ζ-potential indicate that fimbria does not alter the cell’s surface-charge.

MF-SCD and MF–SCDpol are also negatively charged (ζ~–7 mV and –18 mV, respectively). Indeed, there was slight growth of the bacteria’s ζ-potential 5–8 mV after the incubation with the CD_torus_ in 5–10-fold molar excess. This effect might be examined by the shielding of the cell’s surface-charge by carriers, due to their adsorption.

### 2.5. The Adsorption of MF-SCD Complexes on E. coli Cells

**Figure 8:**
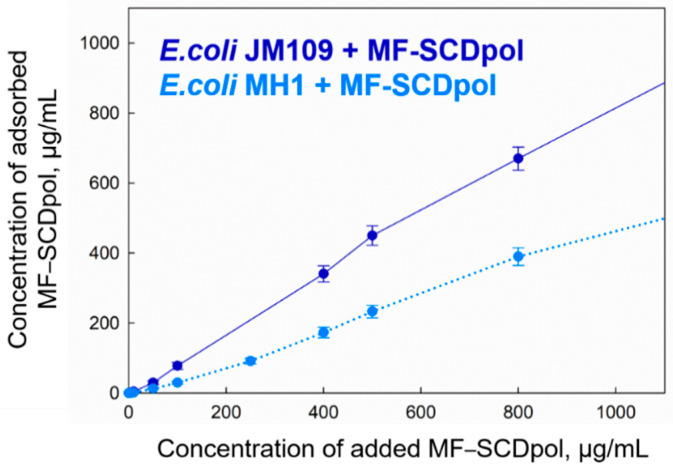
The absorption isotherms of MF–SCDpol on the *E. coli* MH1 and *E. coli* JM109 strains, ~1 × 10^9^ CFU/mL, sodium phosphate buffer solution, pH 7.4, 1 h of incubation at 37 °C.

For a more in-depth study, we obtained the adsorption isotherms, which are presented as the dependence of C_MFabsorbed_ on the C_MFadded_ (Figure 8). The concentration range was chosen in line with our antibacterial study in liquid media (<1 μg/mL). We compared the slopes of the linear curves for MF, MF−SCD and MF−SCDpol. MF–SCD complex formation increased MF absorption by 2–3 times, while SCDpol carrier increased C_MFadsorbed_ by 4.5–5.5 times. Moreover, *E. coli* absorbed 50–90% of the added MF–SCDpol (Figure 8). Moreover, the *E. coli* JM109 strain had a 2–3-times higher affinity for the MF–SCDpol than the *E. coli* MH1.

### 2.6. The Visualization of Particle Adsorption by TEM

The SCDpol absorption was challenging, since organic nanoparticles lack the heavy atoms that can effectively scatter the electrons. Moreover, the low stability of some samples under the electron beam hindered the use of TEM. What is more, in our case, SCDpol and its complex with MF are highly hydrophilic particles that poorly adhere to the support films. In the literature to solve these problems, organic−metal complexes are obtained [36] or drugs with a hydrophobic fragment are used, along with sodium phosphotungstate [37].

CDs interact with a number of metal ions, forming supramolecular homo- and heterometal structures (for example, sandwiches, cylinders, zig-zag patterns, and ext., Figure S5a). Metal ions are coordinated by CD’s –OH groups. Moreover, the structures are maintained by intramolecular hydrogen bonds [38]. In the case of SCDpol, there might also be electrostatic interactions between the sulfo groups and Cu^2+^ ions (Figure S5b). Thus, to obtain the micrographs of the CD-particles, we contrasted the organic matrix with the specific reaction with copper (II) ions. This approach has recently demonstrated the improvement of 2-hydroxypropyl-CD based polymer (HPCDpol) visualization by TEM in our previous work [39].

**Figure 9:**
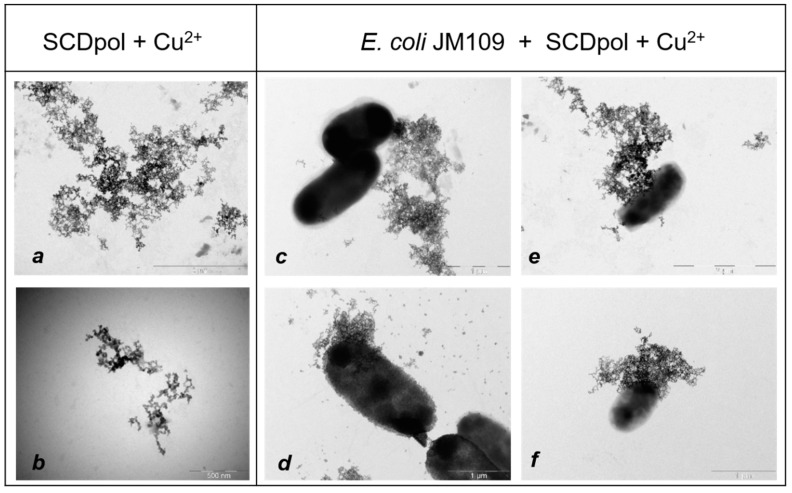
The TEM microphotographs of the SCDpol+Cu^2+^ particles (**a**,**b**) and their absorption on the *E. coli* JM109 cells (**c**–**f**), H_2_O, 1 h of incubation, 37 °C, C_(Cu2+)_ < 0.01 M, 80 mV. Scale bars—2 μm, 500 nm, 1 μm, 2 μm and 1 μm, respectively.

The contrast of SCDpol indeed allowed us to obtain the micrographs of the particle’s aggregates (SCDpol + Cu^2+^) (Figure 9a,b).

Under these conditions, the cells appeared as black oval shapes (Figure 9), as was shown previously for different bacterial strains [39,40]. The presence of copper (II) ions (C_(Cu2+)_ < 0.01 M) did not lead to observable changes in the shape of the cells. Figure 9c–f demonstrates the adhesion of the SCDpol + Cu^2+^ on the bacteria surface. According to the results, particles do not demonstrate selectivity to the specific binding site, but are distributed evenly over the cell surface.

## 3. Discussion

In vitro studies of drug-delivery systems with prolonged release are focused on the modulation in the drug’s MIC or on its antibacterial effect at 24–48 h [41,42,43]. However, numerous carriers demonstrate an extremely slow release-rate: 100% release is reached on the ninth or tenth day [13,15]. Thus, applied technics cannot be used to uncover all the antibacterial properties of drug forms.

Indeed, classical microbiological methods have time limits that are mainly associated with fast bacteria-growth [12]. Nevertheless, there are some long-term studies using bacteria. For instance, Taha M. et al. [14] investigated the antibacterial activity of implants covered with a polymer based on methyl-β-CD, loaded with tobramycin or rifampicin. The disk samples were soaked in buffer, and the supernatant aliquots were tested against the *Staphylococci* and *Enterobacter* species for 14–21 days. However, the study was conducted daily on a bacterial culture that was in the same growth-phase. This means that the authors did not demonstrate the antibacterial effect via the microorganism growth-dynamics (for several days), which is more consistent with the real pathogen state in vivo.

Hence, we developed an in vitro approach to assess the antibacterial activity of drug-delivery systems with prolonged release at an extended period (seven days) on two strains of the gram-negative bacteria *E. coli*. This comprehensive strategy included the selection of physical-chemical (drug-carrier charge, size, etc.) and microbiological (drug concentration, cell-surface morphology) parameters, as well as the control of the bacteria state, so one could observe the impressive increase in the drug’s antibacterial action.

We started with classical microbiological methods in solid and liquid broth-media. MF demonstrated a concentration-dependent antibacterial efficiency (Figure 2 and Figure 4), which is well-known for other antimicrobial agents, for example, clarithromycin [6], azithromycin [29], and nanoparticles of metal oxides [44]. We demonstrated that MF stops working at MIC and 3MIC after 3–4 days of drug exposure. This effect might be explained by the photo- and biodegradation of the fluoroquinolone by the cleavage of the fluorine, oxidation, and/or damage of the drug’s heterocycle [45,46,47]. The change in pharmacophore structure might lead to the absence of the drug’s activity at low concentrations after 3–4 days. Thus, we conclude that the study of the differences between the free drug and the drug incorporated into the DDS should be conducted at a near-MIC concentration, so that the tendency of prolonged antibacterial activity might be examined.

The impact of CD on antimicrobial action was shown earlier for triclosan [8] and oil compounds [9]. The CD polymers’ in vitro activity has not been studied in depth. In our case, SCD and SCDpol increased the antibacterial activity of MF, and here we demonstrated the effect for a longer period. Moreover, for MF-SCDpol we observed the tendency for prolonged antibacterial action that has not yet been described in the literature. These effects might be associated with an increase in the drug solubility and an improvement in the MF adsorption on the cell surface, as well as the protection of drug molecules from biodegradation. Moreover, CDs might cause defects in the lipid membrane [11] and, therefore, increase cell permeability for MF.

During long-term drug exposure, it is important to monitor and understand the impact of our systems (especially carriers) on the cell state, viability, and percentage of dividing cells during drug exposure. We conducted ultrastructural studies using transmission electron microscopy (TEM) to uncover the changes in the cell wall, membrane, and cytoplasm [15,48]. Both *E. coli* strains retained typical structural features, which indicates their viability and the possibility of the long-term experiment up to 7 days (Figure 6). None of the samples caused changes in the bacteria morphology of the cell wall, membrane, or cytoplasm, so SCD and SCDpol do not change MF’s mechanism of action (penetration into the cell by passive diffusion and inhibition of DNA–gyrase [49]).

The increase in drug action by the incorporation of MF in CDs might be explained with other reasons. We consider that the adsorption of the particles on the bacteria surface significantly contributes to the improvement of the MF antibacterial activity.

As was discussed earlier, we chose *E. coli* bacteria with different cell-surface properties (Table S1). We confirmed that *E. coli* JM109 has F-plasmids encoding type 1 pili [50]. The fimbria mainly helps the bacteria to adhere and transfer genetic information [51]. In our case, fimbriae might also confer additional protection against the drug, as the MF’s MIC_JM109_ is ten-fold higher compared with that of MIC_MH1_ (Figure 2), and increases the absorption of negatively charged particles (MF–SCDpol) on the bacteria surface (Figure 8).

Unexpectedly, the negatively charged bacteria [52,53] absorb anionic polymers, and the fimbriae have a great impact on the mechanism of adsorption. For example, Stenström A.T. and Kjelleberg S. [54] studied *S. typhimurium* strains and discovered that bacteria with fimbriae demonstrate higher cell-adhesion on the mineral particles with ζ-potential −14–−40 mV than the strain that lacks fimbriae. To explain this effect, it is worth discussing the amino acid composition of *E. coli* pili [55]: alanine, aspartic acid, threonine, valine, glycine and glutamic acid form approximately 60 mol% of *E. coli* 346 pili. The presence of positively charged amino acids, for instance lysine and histidine, is less than 5 mol%; fimbria is supposedly hydrophobic and negatively charged. Nevertheless, the authors [54] explain the high adhesion of cells with fimbriae on anionic surfaces by the ability of the fimbriae to expose their positively charged groups and hydrophobic fragments to overcome the force of the bacteria’s repulsion. Thus, we consider that the most significant effect of the increase of antibacterial activity and prolonged antibacterial action for negatively charged particles can be demonstrated on the bacteria with fimbriae.

Moreover, the anionic polymers (mainly chelating agents [56]) increase the susceptibility of the bacterial cells, due to the interactions with divalent ions (Ca^2+^ and Mg^2+^) that stabilize lipopolysaccharides in the outer membrane for gram-negative bacteria, and teichoic acids for gram-positive bacteria [29].

Thus, we suggest that MF-carrier adsorption on bacteria contributes to the increase in MF antibacterial activity in DDS by the interaction with the bacteria surface. Nevertheless, the occurrence of severe defects on the bacterial surface is unlikely, since we did not observe any in the ultrastructural studies (Figure 6).

Absorption is an important factor in the increase of antibacterial activity of DDS. For SCDpol’s adsorption visualization, we contrasted the polymer matrix with copper (II) ions. SCDpol + Cu^2+^ aggregates covered the bacteria surface (Figure 9c–f). The adhesion was quite strong, and the polymer almost «enveloped» the bacterial cell (Figure 9d). Similar patterns were demonstrated by the authors of [40], who studied TiO_2_ adsorption on *E. coli* K-12. Moreover, the data correlated with our recent research of the 2-hydroxypropyl-CD-based polymer (HPCDpol) [39]: HPCDpol + Cu^2+^ particles adhered evenly on *Lactobacillus fermentum* and *Bacillus subtilis* ATCC 6633. Nevertheless, at the same concentrations, SCDpol demonstrated higher adsorption on the bacteria surface than HPCDpol.

The obtained micrographs prove that CD-particle adsorption might be one of the main factors that increases the drug’s activity, in conjunction with the polymer matrix.

## 4. Materials and Methods

### 4.1. Reagents and Bacterial Strains

The fluoroquinolone moxifloxacin (MF) was from Kanonpharma (Moscow, Russia). The sulfobutyl ether β-cyclodextrin sodium salt (SCD) was from Zibo Qianhui Biotechnology Co. (Zibo, China). The 1,6-hexamethylene diisocyanate (HMD) and dimethyl sulfoxide (DMSO) were both from Sigma-Aldrich (Saint Louis, MO, USA). The sodium phosphate buffer tablets for the PBS buffer preparation were from Pan-Eco (Moscow, Russia). Hydrochloric acid, copper (II) sulfate, ethanol, and acetone were all purchased from Reakhim (Moscow, Russia). The lead citrate was from Serva (Heidelberg, Germany). Sodium cacodylate, osmium tetroxide, and phosphor-tungstic acid were all from SPI (Anaheim, CA, USA).

Both gram-negative bacterial strains, *Escherichia coli* MH1 (M. Hall, New York, NY, USA [57]) and *Escherichia coli* JM109 (J. Messing, Minneapolis, MN, USA [58]) were kindly provided by Dr. A. Belogurov (National Medical Research Centre of Cardiology, Moscow, Russia).

### 4.2. Synthesis of SCDpol

The synthesis of SCDpol was performed as per [23]. Briefly, to aqueous SCD solution (2 mM, pH 4.0, 37 °C), HMD (0.1 M in DMSO) was added, dropwise. The molar ratio of HMD: SCD was 5:1, and the H_2_O: DMSO ratio was maintained at 1:1 *v*/*v*. The reaction mixture was intensely stirred for 2 h at 25 °C. The removal of the organic solvent was conducted by dialysis, using a membrane with MWCO 3.5 kDa (Serva) for 24 h at 37 °C. The samples were lyophilized for 72 h at −60 °C, using a freeze-dryer Thermo Scientific (Waltham, MA, USA).

### 4.3. Complex Formation of MF with CD Derivatives

MF-CD complexes were obtained by mixing CD and MF solutions (both in 0.1 mM HCl, pH 4.0). C_MF_ was maintained at 1 mg/mL, and the molar ratio of MF: CD torus was 1:1. The complexes were incubated for 1 h at 37 °C, under intense stirring. After this, the samples were lyophilized for 72 h at −60 °C, using a freeze-dryer Thermo Scientific (USA).

### 4.4. Dynamic Light Scattering

The ζ-potential of the sample (in MilliQ H_2_O) was conducted using dynamic light scattering (DLS), using Zetasizer Nano S (Malvern Panalytical, Malvern, UK). The device was equipped with a 4 mW He–Ne-laser (633 nm) and a thermostatic cell (25 °C). We used the Correlator system K7032-09 (Malvern, Worcestershire, UK) and software «Zetasizer Software, version 7.12» (Nanosight Ltd., Salisbury, UK) for data processing. The measurements were performed three times, and the values were reported with standard deviations.

### 4.5. Nanoparticle Tracking Analysis

The SCDpol solution was diluted using Milli Q-purified water to obtain C_particles_ = 10^7^–10^9^ particles/mL. Nanoparticle tracking analysis (NTA) was conducted using the Nanosight LM10-HS instrument (Nanosight Ltd., Salisbury, UK). The measurements were performed three times, and the values were reported with standard deviations.

### 4.6. Fluorescence Spectroscopy

MF emission spectra were recorded within a 400–550 nm range at λ_ex_ = 287 nm in a thermostatic cell (25 °C), using a Varian Cary Eclipse (Santa Clara, CA, USA). The analysis of C_MF_ was performed using the calibration curve (λ_max_ = 465 nm).

### 4.7. In Vitro Experiments: Growth Conditions, Antibacterial Activity Study, Surface Morphology Study, Ultrastructural Studies of Bacteria

For all in vitro experiments, we cultured bacteria in a Luria–Bertani (LB) liquid medium for 12 h (overnight culture). The determination of the minimum inhibition concentration (MIC) was performed in solid media using the agar-well diffusion method [12]. Briefly, the bacteria suspension was distributed over the agar surface on Petri dishes. After 20 min, five discs were incised using a sterile plastic-pipette tip (d = 9 mm). The wells were filled with 50 μL of the samples. After 20 min, the Petri dishes were placed into the incubator at 37 °C for 24 h. Then, the inhibition zone diameters (DIZ) were measured. We considered the MIC value as the sample concentration at which DIZ = 10 mm.

The experiments in liquid media were conducted using double diluted cell-culture (overnight culture was diluted with the growth medium). A total of 1.8 mL of bacteria culture was placed into 5 mL sterile Eppendorf tubes. Then, 0.2 mL of the sample (PBS buffer, MF, MF-SCD or MF-SCD), was added. The specimens were incubated at 37 °C and 100 rpm stirring for 7 days. At the specific time, 100 μL of each sample was diluted, and its absorption was measured at 600 nm, using the Ultrospec 2100 pro instrument (Biochrom, Holliston, MA, USA). For quantitative analysis, 50 μL of each sample was diluted and seeded on the Petri dish. The plates were placed in the incubator at 37 °C. After 24 h, the number of colony-forming units (CFU) was analyzed. The antibacterial activity (AA) of the drug was determined according to the formula:

$$\mathrm{AA}=\mathrm{lg}\frac{{\mathrm{CFU}}_{\mathrm{control}}}{{\mathrm{CFU}}_{\mathrm{MF}}}$$

where CFU_control_ and CFU_MF_ are the number of CFU in the control culture (PBS buffer) and the sample that contained MF, respectively. The effect of MF-CD complex formation on the drug’s antibacterial action was determined as drug’s action efficiency (DAE):

$$\mathrm{DAE}=\frac{{\mathrm{CFU}}_{\mathrm{MF}}}{{\mathrm{CFU}}_{\mathrm{complex}}}$$

where CFU_MF_ and CFU_complex_ are the number of CFUs in the sample containing free MF and MF in the MF-CD complex, respectively. In vitro experiments were independently performed three times, and average values were reported with standard deviations.

The preparation of the bacteria culture for the TEM microphotographs was performed as per the studies in [15,39]. Briefly, the 900−1000 μL of bacterial culture was centrifuged (Eppendorf Minispin, 7500 rpm/min, 7 min, Hamburg, Germany). The pelleted cells were fixed with OHC(CH_2_)_3_CHO solution (2.5% in 100 mM sodium cacodylate (SC)), for 24 h. The pellets were rinsed 3 times (each for 5 min) in 100 mM SC, under slight agitation. Then, the samples were fixed with OsO_4_ (1% in 100 mM SC) for 1 h at +4 °C. The fixed cells were dehydrated with EtOH (raised concentrations: 50%−70%−96%). Then, 96% EtOH was replaced with acetone, and then with the mixtures of epoxy resin (SPI-Pon 812, SPI Supplies (Sigma-Aldrich)) and acetone, with an increasing resin concentration until pure resin was obtained. The samples were stored at +70 °C for 48 h. Ultrathin sections (nominal thickness = 80 nm) were cut using a Reichert-Jung Ultracut E ultramicrotome, equipped with a Diatome Ultra 45 diamond knife. The sections were mounted on the formvar-coated Cu slot-grids, and poststained with lead citrate for 3 min.

For the cell-surface study, ~15–20 μL drops of the bacterial culture were placed on the formvar-coated 200-mesh Cu grids for 60–90 s. Then, the drops were blotted with the filter paper. After that, the 10 μL of aqueous tungstophosphoric acid (1%) was applied onto the grids for 20 s, and blotted with the filter paper. Finally, the samples were air-dried. TEM microphotographs were obtained and analyzed using a JEM-1400 electron microscope (Jeol, Tokio, Japan) running at 80 kV, and equipped with a Quemesa digital camera (OSIS, Münster, Germany).

### 4.8. The Particle Adsorption on Bacteria

The study of SCDpol adsorption on bacteria was conducted as performed by the authors in [39]. The 900 μL of bacterial overnight culture was centrifuged (Eppendorf Minispin, 7000 rpm/min, 5 min) and was washed using sterile sodium phosphate buffer-solution with pH 7.4 (3 times for each 1 mL). The MF-SCDpol solutions were added to the bacteria (~8 × 10^8^ CFU/mL), and mixed properly. The samples were incubated for 1 h at 37 °C and 100 rpm. Then, they were centrifuged (7000 rpm/min, 5 min), and the supernatant was studied using fluorescence spectroscopy, to analyze the free MF-SCDpol.

## 5. Conclusions

We propose a new method for studying antibacterial-drug activity in vitro for a long-time period (7 days). This strategy is crucial for uncovering the tendency of prolonged antibacterial action of the drug incorporated into DDS, before studies using eukaryotic cells and in vivo studies. Through the complex physical-chemical methods and classical microbiology approaches, we demonstrate the antibacterial action and the ultrastructural morphology of two *E. coli* strains for 7 days. At concentrations near MIC values, MF loses its antibacterial action, whereas DDS based on CDs improves the drug action. Interestingly, the morphology of the bacteria surface has an important role in particle adhesion: the fimbriae increase the particle adsorption by almost two times. We believe the suggested new approach will help other researchers to understand the complete potential of the drug’s antibacterial action in DDS. For instance, the observed tendency for prolonged antibacterial action might cause a decrease in the drug dosage or a decrease in the number of times the drug is administered. The strategy is promising for deep in vitro study of new drugs and novel drug forms, especially those which demonstrate sustained drug release.

## Figures and Tables

**Table 1:** Physical-chemical characteristics of SCD and SCDpol.

CD	Hydrodynamic Diameter, nm	ζ-Potential, mV	M, kDa	K_dis_ of MF-CD, M
SCD	~0.15	−7.3 ± 0.8	~2.1	1.0 (±0.3) × 10^−4^
SCDpol	190 ± 15	18.7 ± 1.1	140 ± 14	5.2 (±0.3) × 10^−6^

## Data Availability

Not applicable.

## Supplementary Materials

The following supporting information can be downloaded at: https://www.mdpi.com/article/10.3390/molecules27228026/s1, Figure S1: The raw data of appeared inhibition zones on Petri dishes, agar diffusion method, C_MF_ = 0.05–10 μg/mL, pH 7.4 (0.01M PBS), 37 °C, 24 h of incubation, Figure S2: The number of colony-forming units (bars) and the absorbance at 600 nm (lines) of the control *E. coli* MH1 culture (black/grey) and culture incubated with SCDpol (blue), 37 °C, 1–7 days of incubation, Figure S3: The ultrastructure of the overnight control culture *E. coli* MH1 and *E. coli* JM109. Scale bar–2 μm, Figure S4: The ultrastructure of the control culture *E. coli* MH1 after 72 h of incubation. Some of the living (green), dead (red) and dividing (blue) cells are marked by arrors, 37 °C. Scale bar–1 μm, Figure S5: (a) CD-Cu^2+^ complex. (b) Proposed additional electrostatic interactions between SCDpol and copper (II) ions [38], Table S1: E. coli strains and genotypes [57,58,59].

## Sample Availability

Samples of the compounds are available from the authors.
