# Supplementary material for: The New Strategy for Studying Drug-Delivery Systems with Prolonged Release: Seven-Day In Vitro Antibacterial Action
Source: Molecules. 2022 Nov 18;27(22):8026. doi: 10.3390/molecules27228026 (PMC9695913; doi:10.3390/molecules27228026)
Supplement: Supplementary file 1 [file molecules-27-08026-s001.zip › molecules-2020236-supplementary.pdf]

molecules

Article

The New Strategy for Studying Drug-Delivery Systems with Prolonged Release: Seven-Day In Vitro Antibacterial Action

Anna A. Skuredina [1], Tatiana Yu. Kopnova [1], Anastasia S. Tychinina [1], Sergey A. Golyshev [2], Irina M. Le-Deygen [1], Natalya G. Belogurova [1] and Elena V. Kudryashova [1,*]

Supplementary

Table S1 *E. coli* strains and genotypes [57–59].

E. coli MH1	F *araD139 Δ(lac)X74 galU galK hsdR2 mcrB1 rpsL*
E. coli JM109	*recA1 endAl gyrA96 thi hsdR17 supE44* relA1 I-<br>*Δ(lac-proAB)* F' *traD36 proAB lacZΔM15*

Figure S1 The raw data of appeared inhibition zones on Petri dishes, agar diffusion method, C_{MF}= 0.05 – 10 μg/mL, pH 7.4 (0.01M PBS), 37°C, 24 h of incubation.

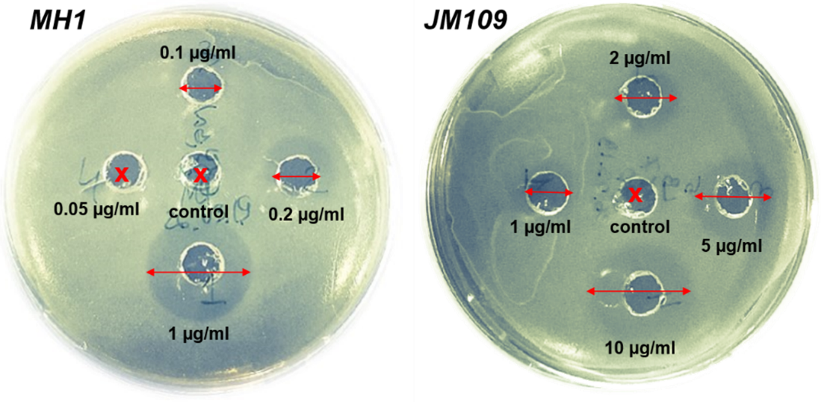

Figure S2 The number of colony-forming units (bars) and the absorbance at 600 nm (lines) of the control *E. coli* MH1 culture (black/grey) and culture incubated with SCDpol (blue), 37°C, 1-7 days of incubation.

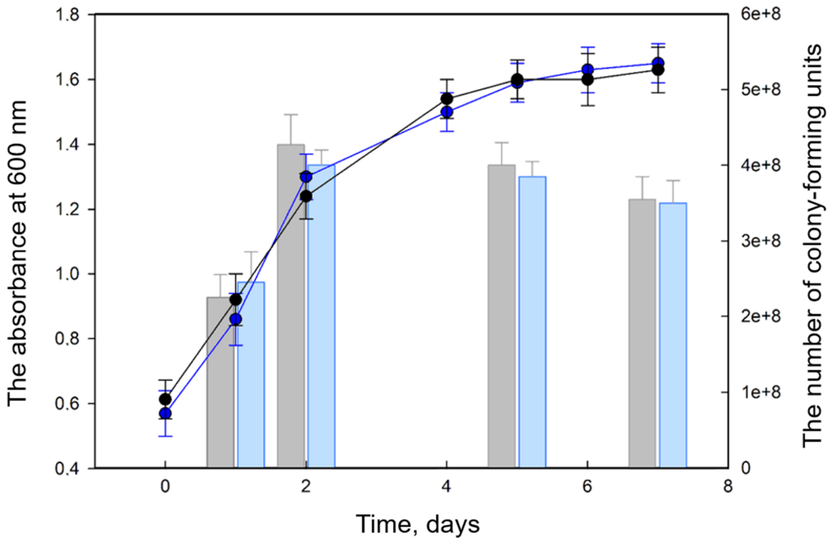

Figure S3 The ultrastructure of the overnight control culture *E. coli* MH1 and *E. coli* JM109. Scale bar - 2 μm.

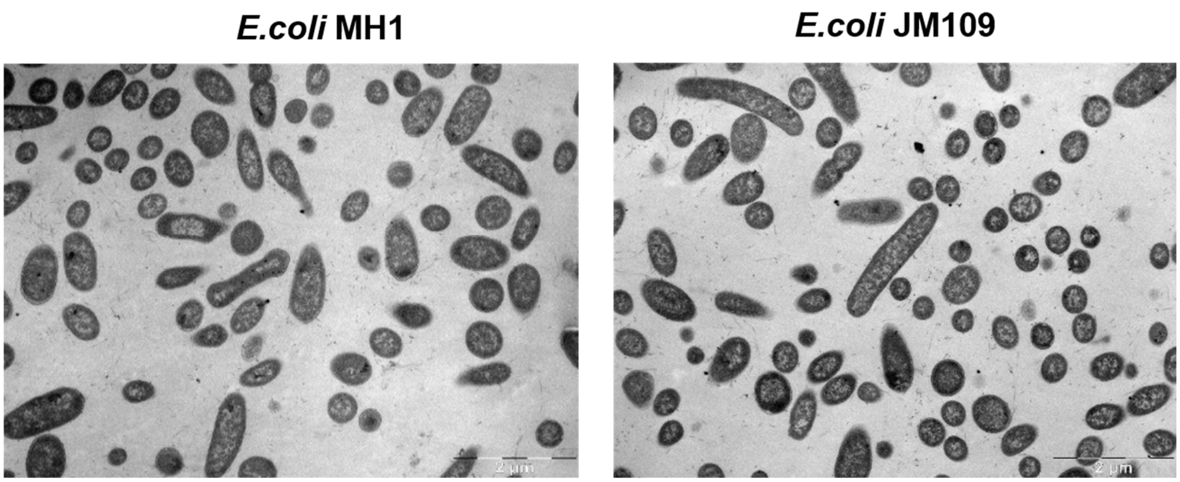

Figure S4 The ultrastructure of the control culture *E. coli* MH1 after 72 hours of incubation. Some of the living (green), dead (red) and dividing (blue) cells are marked by arrors, 37°C. Scale bar - 1 μm.

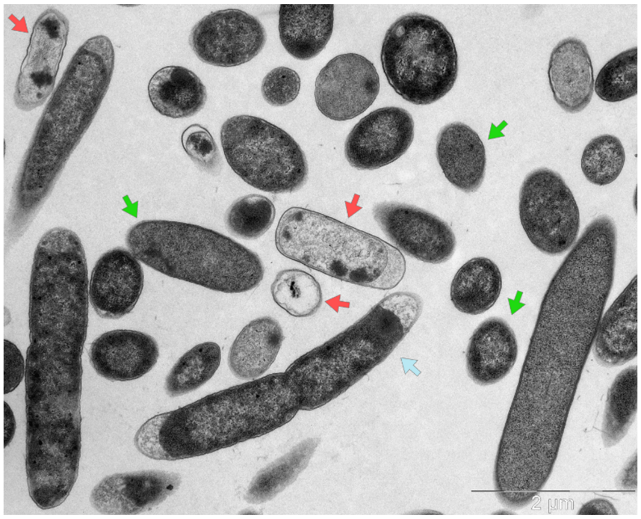

Figure S5 (a) CD-Cu^{2+} complex [38]. (b) Proposed additional electrostatic interactions between SCD-pol and copper (II) ions.

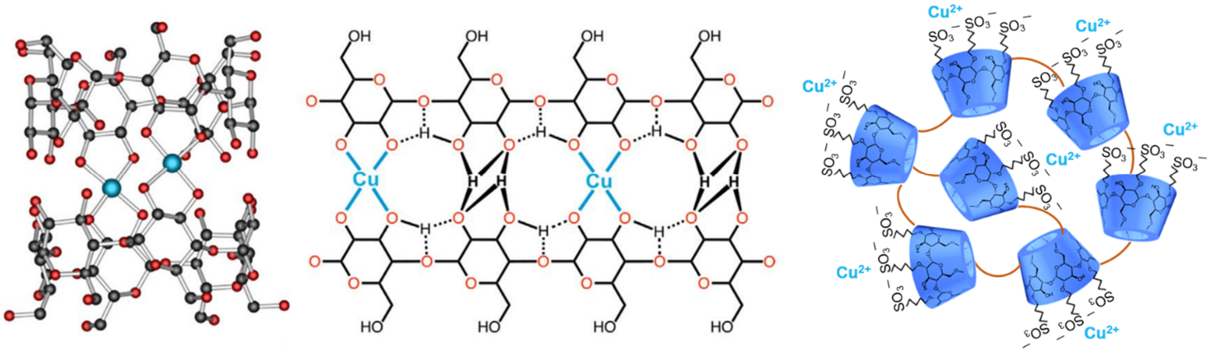

(a) (b)
